# Supplementary material for: Coping self-efficacy and social support as predictors of adolescent sex trafficking exit: Results of a secondary analysis
Source: PLoS One. 2024 Jan 2;19(1):e0291207. doi: 10.1371/journal.pone.0291207 (PMC10760720; doi:10.1371/journal.pone.0291207)
Supplement: Attachment — Submitted filename: Response to Reviewers.docx [file pone.0291207.s005.docx]

August 23, 2023

To the members of the *PLOS ONE* editorial board*,*

I am pleased to resubmit my manuscript to you, titled *Coping Self- Efficacy and Social Support as Predictors of Adolescent Sex Trafficking Exit: Results of a Secondary Analysis.* The revisions required by the reviewers and editors were relatively minimal, so we have completed them as requested. Please see the below summary of the changes we have made to the original manuscript in the revision, with requested revisions included in quotation marks:

*Reviewer 1:*

1. “In introduction section, give some statistics of victims/cases/incidents of the problem (if any) globally as well as locally where the study was conducted in order to clearly understand the severity of the problem.”

Although we understand the importance of contextualizing incidence/prevalence, we hesitate to add this information to the manuscript because CSEY prevalence statistics are widely understood within the field as inaccurate and unreliable. Responsibly discussing prevalence rate data would require the addition of several paragraphs of discussion, which we feel would detract from the overall purpose of the paper and its focus on CSEY exit. For instance, in the protocol that we published in PLOS ONE prior to the current manuscript, we cited national and state data, but had to explain how one data source cited only ~670 CSEY cases in Texas while another data source suggested there are 79,000 CSEY victims in Texas. Since the estimate range is so high and requires significant explanation, we opted instead to add the following sentence to the introduction section: “It is important to investigate CSEY survivors’ service outcomes because revictimization and re-trafficking are common [13] and the hidden nature of the population makes them hard to research [14,15] **resulting in few reliable estimates of CSEY prevalence**, and few studies on whether social work case management services effectively promote an exit from CSE.”

*Reviewer 2:*

1. “Good effort and you have presented all the data in proper way. It is very informative but if you conduct this study by taking the primary data it would more beneficial. I suggest you can conduct primary study on this topic in future.”

We have added a statement about the importance of conducting similar research with primary data in the conclusion section of the paper.

*Editors:*

1. “Please ensure that your manuscript meets PLOS ONE's style requirements, including those for file naming.”

We have reviewed the file naming requirements for PLOS ONE and believe that the files match the requirements. Please let us know if any additional changes are required.

2. “Thank you for stating the following in the Acknowledgments Section of your manuscript: ‘This study was made possible by the support of the Office of the Texas Governor’s Child Sex Trafficking Team and Traffick911.’ However, funding information should not appear in the Acknowledgments section or other areas of your manuscript. We will only publish funding information present in the Funding Statement section of the online submission form. Please remove any funding-related text from the manuscript and let us know how you would like to update your Funding Statement. Please include your amended statements within your cover letter; we will change the online submission form on your behalf.”

We have removed the statement about funding from the Acknowledgements section of the manuscript, instead replacing it with an acknowledgement of Traffick911’s logistical support for the project. We have also reviewed the manuscript to make sure this information doesn’t appear anywhere else within the document.

The Funding Statement can remain as follows within the PLOS ONE system: “This project was supported by a Victims of Crime Act (VOCA) federal pass-through grant awarded to the Office of the Texas Governor (OOG) Child Sex Trafficking Team and Traffick911. The sponsor of this protocol – Traffick911 – has assisted with the study design and data collection plan described in this protocol. Traffick911 administrators have approved of the protocol described herein. The original funders of this project, including VOCA and the OOG, had no role in study design, data collection and analysis, decision to publish, or preparation of the manuscript. The opinions, findings, conclusions, and recommendations expressed in this publication are those of the authors and do not necessarily reflect the views of VOCA, OOG, or Traffick911. ([www.traffick911.com)](http://www.traffick911.com)).”

3. “In your Data Availability statement, you have not specified where the minimal data set underlying the results described in your manuscript can be found. PLOS defines a study's minimal data set as the underlying data used to reach the conclusions drawn in the manuscript and any additional data required to replicate the reported study findings in their entirety. All PLOS journals require that the minimal data set be made fully available. For more information about our data policy, please see <http://journals.plos.org/plosone/s/data-availability>. Upon re-submitting your revised manuscript, please upload your study’s minimal underlying data set as either Supporting Information files or to a stable, public repository and include the relevant URLs, DOIs, or accession numbers within your revised cover letter. For a list of acceptable repositories, please see http://journals.plos.org/plosone/s/data-availability#loc-recommended-repositories. Any potentially identifying patient information must be fully anonymized. Important: If there are ethical or legal restrictions to sharing your data publicly, please explain these restrictions in detail. Please see our guidelines for more information on what we consider unacceptable restrictions to publicly sharing data: http://journals.plos.org/plosone/s/data-availability#loc-unacceptable-data-access-restrictions. Note that it is not acceptable for the authors to be the sole named individuals responsible for ensuring data access. We will update your Data Availability statement to reflect the information you provide in your cover letter.”

We have added a minimal underlying dataset to the submission as “S4_File”, and also added a brief statement about this data in the “Supporting Information” section of the manuscript.

4. “Please review your reference list to ensure that it is complete and correct. If you have cited papers that have been retracted, please include the rationale for doing so in the manuscript text, or remove these references and replace them with relevant current references. Any changes to the reference list should be mentioned in the rebuttal letter that accompanies your revised manuscript. If you need to cite a retracted article, indicate the article’s retracted status in the References list and also include a citation and full reference for the retraction notice.”

We have reviewed the references list and believe that it is complete and accurate.

We look forward to hearing back from you about the manuscript. Thank you for your time and attention to the manuscript.

Sincerely,

Mary K. Twis, PhD, LMSW-AP

Assistant Professor

TCU Department of Social Work

Mary.twis@tcu.edu
